# HER2-HER3 dimer quantification by FLIM-FRET predicts breast cancer metastatic relapse independently of HER2 IHC status

**DOI:** 10.18632/oncotarget.9963

**Published:** 2016-07-07

**Authors:** Gregory Weitsman, Paul R. Barber, Lan K. Nguyen, Katherine Lawler, Gargi Patel, Natalie Woodman, Muireann T. Kelleher, Sarah E. Pinder, Mark Rowley, Paul A. Ellis, Anand D. Purushotham, Anthonius C. Coolen, Boris N. Kholodenko, Borivoj Vojnovic, Cheryl Gillett, Tony Ng

**Affiliations:** ^1^ Richard Dimbleby Department of Cancer Research, Randall Division and Division of Cancer Studies, King's College London, Guy's Medical School Campus, London, UK; ^2^ Department of Oncology, Cancer Research UK and Medical Research Council Oxford Institute for Radiation Oncology, University of Oxford, Oxford, UK; ^3^ Institute for Mathematical and Molecular Biomedicine, King's College London, Guy's Medical School Campus, London, UK; ^4^ Research Oncology, Division of Cancer Studies, King's College London, Guy's Hospital, Great Maze Pond, London, UK; ^5^ Breakthrough Breast Cancer Research Unit, Department of Research Oncology, Guy's Hospital King's College London School of Medicine, London, UK; ^6^ UCL Cancer Institute, Paul O'Gorman Building, University College London, London, UK; ^7^ Systems Biology Ireland, University College Dublin, Belfield, Dublin, Ireland; ^8^ Department of Medical Oncology, St George's Hospital NHS Foundation Trust, London, UK; ^9^ Sussex Cancer Centre, Brighton and Sussex University Hospitals, Royal Sussex County Hospital, Brighton, UK; ^10^ Department of Biochemistry and Molecular Biology, School of Biomedical Sciences and Biomedical Discovery Institute, Monash University, Melbourne, Australia

**Keywords:** breast cancer, HER2, HER3, FLIM-FRET, prognosis

## Abstract

Overexpression of HER2 is an important prognostic marker, and the only predictive biomarker of response to HER2-targeted therapies in invasive breast cancer. HER2-HER3 dimer has been shown to drive proliferation and tumor progression, and targeting of this dimer with pertuzumab alongside chemotherapy and trastuzumab, has shown significant clinical utility. The purpose of this study was to accurately quantify HER2-HER3 dimerisation in formalin fixed paraffin embedded (FFPE) breast cancer tissue as a novel prognostic biomarker.

FFPE tissues were obtained from patients included in the METABRIC (Molecular Taxonomy of Breast Cancer International Consortium) study. HER2-HER3 dimerisation was quantified using an improved fluorescence lifetime imaging microscopy (FLIM) histology-based analysis. Analysis of 131 tissue microarray cores demonstrated that the extent of HER2-HER3 dimer formation as measured by Förster Resonance Energy Transfer (FRET) determined through FLIM predicts the likelihood of metastatic relapse up to 10 years after surgery (hazard ratio 3.91 (1.61–9.5), *p* = 0.003) independently of HER2 expression, in a multivariate model. Interestingly there was no correlation between the level of HER2 protein expressed and HER2-HER3 heterodimer formation. We used a mathematical model that takes into account the complex interactions in a network of all four HER proteins to explain this counterintuitive finding.

Future utility of this technique may highlight a group of patients who do not overexpress HER2 protein but are nevertheless dependent on the HER2-HER3 heterodimer as driver of proliferation. This assay could, if validated in a group of patients treated with, for instance pertuzumab, be used as a predictive biomarker to predict for response to such targeted therapies.

## INTRODUCTION

The human epidermal growth factor receptors HER2 (ErbB2) and HER3 (ErbB3) are members of the HER family of receptor tyrosine kinases [1]. They are important in the development of cancer and the over-expression of HER2 has been shown to increase the downstream signalling of ERK1/2 and PI3K/Akt leading to tumor promotion [2].

The HER2-HER3 dimer is central to oncogenic proliferation and the HER2 receptor has been used to identify a subgroup of breast cancer patients with poor prognosis, but is specifically also a target for therapy. HER2 overexpression or gene amplification is the only validated predictive biomarkers in use for the stratification of breast cancer patients for targeted therapies such as trastuzumab (HER2-targeted), pertuzumab (a monoclonal antibody targeted against HER2-HER3 dimerisation with other HER receptors), TDM-1 and lapatinib (against both EGFR (HER1) and HER2) [3–5]. Over the last 2 decades, although a variety of HER2-targeted therapies have been developed [6], novel predictive tools for patient selection have failed to be validated. For instance, despite extensive biomarker analyses of samples from the CLEOPATRA study, including serum markers, ligands to HER family members, expression levels of HER family members by mRNA and immunohistochemistry (IHC), and intracellular pathway markers, HER2 protein overexpression determined by IHC or HER2 amplification by fluorescent *in situ* hybridisation (FISH) remained the only predictive marker for patient selection for the trastuzumab and pertuzumab combination [7]. In this clinical trial high levels of HER2 and HER3 mRNA were prognostic of improved progression free survival with modest hazard ratios (HR) of 0.77 and 0.81 respectively. The HR for HER2 membranous expression by IHC was not found to be significant (HR of 0.83 and a *p*-value of 0.05).

These results reinforce the need for more accurate patient stratification beyond the Food and Drug Administration-approved assays that measure HER2 protein expression such as the HercepTest [3, 8], that are used to identify HER2 positive tumors as appropriate for HER2 targeted treatment. The use of additional anti-HER2 dimerization targeted agents in the neoadjuvant setting has demonstrated improved complete pathological response rates (pCR), which led to the postulation that de-escalation of treatment may be a viable option for some patients [9]. For instance, without patient stratification, a combination of the targeted drugs, trastuzumab and pertuzumab, produced a pCR of 16.8% (compared to 45.8% pCR for patients on the targeted drugs plus docetaxel). Similarly patients with metastatic HER2-positive breast cancer may receive combination chemotherapy with trastuzumab and pertuzumab (CLEOPATRA [10, 11]), but there is no evidence for the use of the targeted drugs alone. However, some patients may potentially be able to avoid chemotherapy entirely, whilst patient stratification with HER2 related biomarkers should also lead to increased response rates in those receiving targeted agents plus chemotherapy. In the modern era, the challenge for HER2 positive breast cancer is not only to continue to improve efficacy, but to decrease toxicity and cost, potentially with the development of better biomarkers.

Although there have been major advances in our understanding of the cancer genome on the basis of large scale sequencing efforts such as The Cancer Genomic Atlas (TCGA), International Cancer Genome Consortium (ICGC) and METABRIC, the functional consequences of many recently revealed genomic alterations, including somatic mutations, remain unclear. For HER2, somatic mutations are rarely found but the HER2(YVMA) insertion mutant has been shown to have a higher autocatalytic activity than that of its WT equivalent *in vitro* [12]. Mutations that sensitize tumors to targeted therapies, such as that found in EGFR [13], have so far not been identified for HER2 in the breast cancer setting.

We have, in recent years, used an imaging approach to investigate individual patients' intact tissues at a subcellular and nanometer proximity level, so as to obtain a deeper understanding of the protein complexes and sub-networks, including the HER family, within tumor cells, in order to achieve an improved monitoring of treatment responses and to derive biomarkers that can be used in a patient-specific manner [14–19]. As an example, we recently described the first clinical utilization of a fluorescence lifetime imaging microscopy (FLIM) histology assay to quantify the level of HER1-HER3 dimer formation in formalin-fixed paraffin-embedded (FFPE) basal-like breast cancer tissues from patients treated unsuccessfully with anti-EGFR treatments (cetuximab or panitumumab). Post-treatment biopsies were obtained from patients with residual cancer and compared to pre-treatment samples. An increase in the amount of HER1-HER3 dimer (as part of the HER protein network rewiring) was shown to be a mechanism of anti-EGFR treatment resistance in this neoadjuvant study [14]. We hypothesize that this approach may also be of utility in stratifying patients for HER2 treatment, given the absence of obvious mutations that can report on HER2 sensitivity to targeted therapies, as well as the suboptimal and relatively heterogeneous response rate to molecular therapeutics [20].

As HER3 has been shown to be the preferred interaction partner of HER2 [21, 22] and as pertuzumab is targeted against this interaction, several groups have attempted to quantify HER2-HER3 dimerisation in different cell types and xenograft models [23] as well as in FFPE samples [23, 24]. These studies use either the Proximity Ligation Assay (PLA) or the VeraTag assay, which can both detect proteins in close proximity, inferring interaction. However, for the PLA assay, the maximum distance between antigenic determinants recognized by two antibodies that are directly conjugated to oligonucleotides, has been estimated to be roughly 30 nm, including the size of the two antibodies and the connecting oligonucleotides [25]. The equivalent distance can be up to 300 nm for the VeraTag assay [26, 27]. Using the PLA technique (primary plus secondary antibodies conjugated to oligonucleotides), high levels of HER2-HER2 and HER2-HER3 protein proximity have previously been shown to be correlated with HER2 amplification/overexpression, using 88 and 74 cases of human breast carcinomas for HER2-HER2 and HER2-HER3 PLAs, respectively [24].

We used a combination of fluorescence lifetime imaging microscopy and Förster Resonance Energy Transfer (FLIM-FRET) [16, 18, 28, 29], which has become the accepted gold standard technique for measuring protein proximity, typically within the < 10 nm range (between the centres of the donor and acceptor fluorophores, that label the antibodies), to quantify *in situ* interactions between HER2 and HER3 in FFPE tissue. Part of the difficulty of accurately determining the fluorescence lifetime of fluorophores in FFPE tissue is the presence of interfering endogenous and preparation-induced fluorescence emission in both stromal and epithelial components [30, 31]. These same contaminating “autofluorescence” components also interfere with non-FLIM applications such as confocal laser scanning microscopy (CLSM) although this is often not obvious when intensity is the measured ‘signal’. In order to accurately quantify dimers, we employed our recently improved FLIM histology technique and its associated analysis algorithm [32] derived specifically to circumvent the problem of contaminating fluorescence signals in FFPE tissues. We showed that HER2-HER3 dimer quantification by FLIM-FRET does not correlate with HER2 protein expression by IHC, contrary to the previously reported finding [24]. This was further supported by the lack of correlation between HER2 and HER3 mRNA levels and HER2-HER3 dimer quantification by FLIM-FRET. This novel biomarker predicts breast cancer metastatic relapse independently of HER2 expression, up to 10 years post-surgery, in a multivariate model.

## RESULTS

### Validation of the HER2-HER3 dimer assay

The scoring of HER2 protein immunohistochemical expression patterns in breast cancer patients is a well-established protocol with commercially available validated antibodies (e.g. HercepTest, DAKO), Similar staining intensities and patterns of HER2 localization in tumor cells were obtained using the directly labelled anti-HER2 IgG, compared to those obtained using the conventional IHC method (Supplementary Figure S1A). Alexa546-conjugated anti-HER3-IgG was validated in FFPE cells overexpressing HER3 protein (Supplementary Figure S1B). HER3 expression in tumor samples was assessed and categorised into two groups with either predominantly membrane or cytoplasmic staining, and into a range of different expression levels was seen (Supplementary Figure S1C). Both the pattern and intensity of staining were recorded by a histopathologist (Table 1).

**Table 1:** Clinico-pathological characteristics of patients in METABRIC cohort in king's health partners cancer biobank

		Number (%) of patients *N* = 131	(100%)	Number of patients		
**FRET efficiency**	High (> 8.56%)	53	(40%)	**FRET efficiency High Low**		**Fisher's exact test P**
	Low (< 8.56%)	78	(60%)	25	40	
**HER3 localisation**	C	65	(50%)	13	21	
	C + M	34	(26%)	13	14	0.67
	M/M + C	27	(21%)			
	Not available	5	(4%)	5	17	
**HER2 status (TMA IHC)**	Positive	22	(17%)	38	56	0.15
	Negative	94	(72%)			
	Not available	15	(11%)	47	63	
**ER status**	Positive	110	(84%)	6	15	0.33
	Negative	21	(16%)	27	50	
**PR status**	Positive	77	(59%)	26	28	0.15
	Negative	54	(41%)	17	36	
**Clinical tumour size**	< 20 mm	53	(40%)	36	42	0.15
	> 20 mm	78	(60%)	3	12	
**Histological grade**	1	15	[11%)	17	28	
	2	45	(34%)	30	35	0.18
	3	65	(50%)			
	Not available	6	(5%)	26	25	
**Lymph nodes positive**	0	51	(39%)	17	41	
	1–3	58	(44%)	9	12	0.06
	4+	21	(16%)			
	Not available	1	(1%)	7	17	
**Age**	< 50 yr	24	(18%)	46	61	0.25
	> 50 yr	107	(82%)			
**Menopausal status**	Pre	23	(18%)			
	Post	103	(79%)			
	Peri	4	(3%)			
	Not available	1	(1%)	42	60	
**Treatment (endocrine)**	Yes	102	(78%)	11	18	0.83
[Al/Endo/Endocrine/TAM/TAM;AI]	No	29	(22%)	10	20	
**Treatment (chemotherapy)**	Yes	30	(23%)	43	58	0.40
[APD/Chemo/CMF/Taxoid]	No	101	(77%)			
**Time to distant metastasis**	Number of events = 37					
	Median (event) = 2.7 yr					
	Median (event or last follow-up) = 7.6 yr					

[Al / Endo / Endocrine / TAM / TAM;AI]

[APD / Chemo / CMF / Taxoid]

[None / (no entry)]

[None / (no entry)]

**Figure 1:**
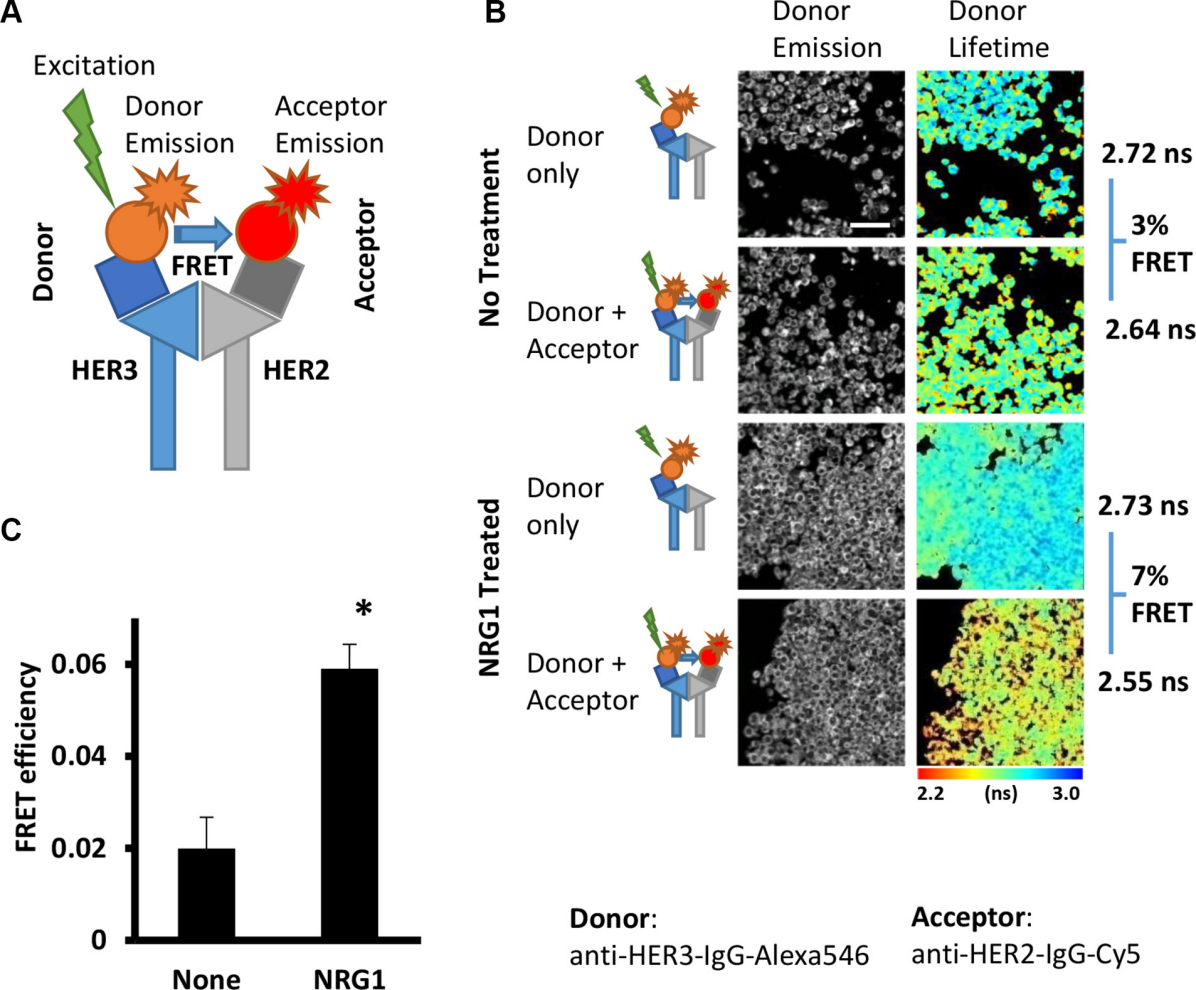
Detection of HER2-HER3 interaction in FFPE cells samples by FRET-FLIM assay (**A**) Schema illustrating the principle of antibody-based FRET-FLIM assay. Energy transfer (FRET) occur between donor fluorophore (Alexa546) and acceptor fluorophore (Cy5) upon excitation of donor only at distance less than 10 nm between fluorophores. (**B**) Interaction between HER2 and HER3 proteins induced by NRG1 treatment. Detected with anti-HER3-IgG-Alexa546 (donor) and anti-HER2-IgG-Cy5 (acceptor) antibodies in FFPE SKBR3 cells. Pseudocolour map shows distribution of measured lifetime where red/yellow pixels represents low lifetime - higher level of HER2-HER3 dimer). Scale bar = 60 μm. (**C**) Quantification of the result presented in A.

Fluorophore-labelled antibodies for the FLIM-FRET assay (Figure 1A) were tested in FFPE SKBR3 cell pellets, mimicking the conditions of tumor tissue fixation and processing. In control cells a low level of interaction was detected between HER2 and HER3 (Figure 1B, small shift to the red end of the spectrum on the pseudo-colour image with the acceptor, Cy5, resulting in a lifetime reduction of Alexa546 (X546) due to FRET between the two proteins in close proximity). Conversely, in cells treated with the HER3 ligand NRG-1 (which causes HER dimer formation [33]), a statistically significant increase in interaction between HER2 and HER3 proteins was obtained (Figure 1C, 2% vs. 6%, *P* = 0.004, marked shift to the red in lifetime image, Figure 1B). Thus the ability of the chosen antibodies to specifically recognize the target proteins and report interaction between them in FFPE samples was confirmed. Validation of this HER2-HER3 FLIM-based dimer assay is being performed in other cell lines (such as colorectal), using a range of stimuli (EGF, NRG1, etc) as well as commonly used anti-EGFR therapeutics (both tyrosine kinase inhibitor and anti-EGFR antibody). Some specificity with respect to ligand requirement (no enhancement with EGF for instance) has been observed (data not presented) in addition to inhibitor-induced HER2-HER3 dimer formation (which is analogous to our previously reported drug-induced EGFR homodimer [34, 35]).

### Measurements of HER2-HER3 dimer levels in human tumors

A total of 152 cores were present on the TMA sections used for FLIM imaging, FLIM images from 131 cores were suitable for use for further analysis and survival modelling. Table 1 demonstrates the tumor characteristics within the FRET ‘high’ and ‘low’ groups (classified using ROC analysis, see materials and methods). There were no significant differences between the 2 groups, but there was a trend to significance for an association with lymph node status: patients with low FRET tumors were more likely to have node positive disease, with the majority having 1–3 nodes involved, compared to those with high FRET tumors, who were more likely to have tumor-free lymph nodes.

**Figure 2:**
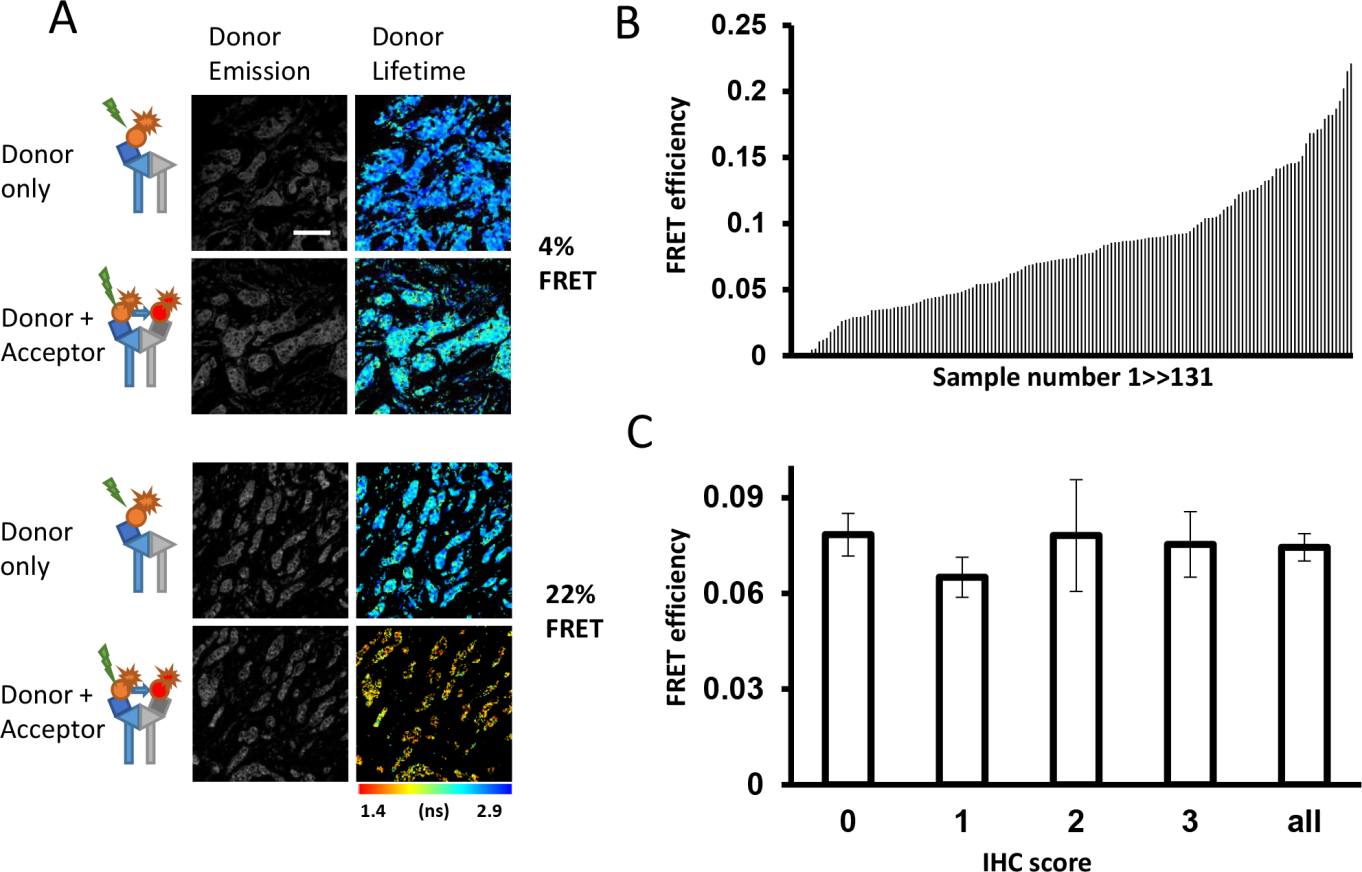
Relationship between HER2-HER3 dimer and expression of proteins in patients' samples (**A**) Representative images of tumors with low (upper panels) and high (bottom panels) levels of HER2-3 interaction. (**B**) Distribution of FRET efficiency signal across patients' tumor cores on Guy's METABRIC TMAs (*N* = 131). (**C**) FRET efficiency (mean ± SEM) shown by available HER2 IHC scores (0,1,2,3; HercepTest) and for all imaged cores.

The measured FRET efficiency ranged from 0% to 22% across the 131 tumor samples (Figure 2A–2B) with an average value of 7.7 ± 0.4% (mean ± SEM, Figure 2C). Since the expression level of HER2 is an established criterion to predict prognosis and drug response of breast cancer [36, 37], the interaction between FRET efficiency (reflecting HER2-HER3 dimer formation) and protein expression of HER2 using standard IHC scoring system was tested.

Surprisingly, in contrast to previously published data with PLA, IHC and FISH techniques [24], no correlation between FRET efficiency and HER2 scoring values derived from traditional IHC staining using HercepTest was observed (Figure 2C).

**Figure 3:**
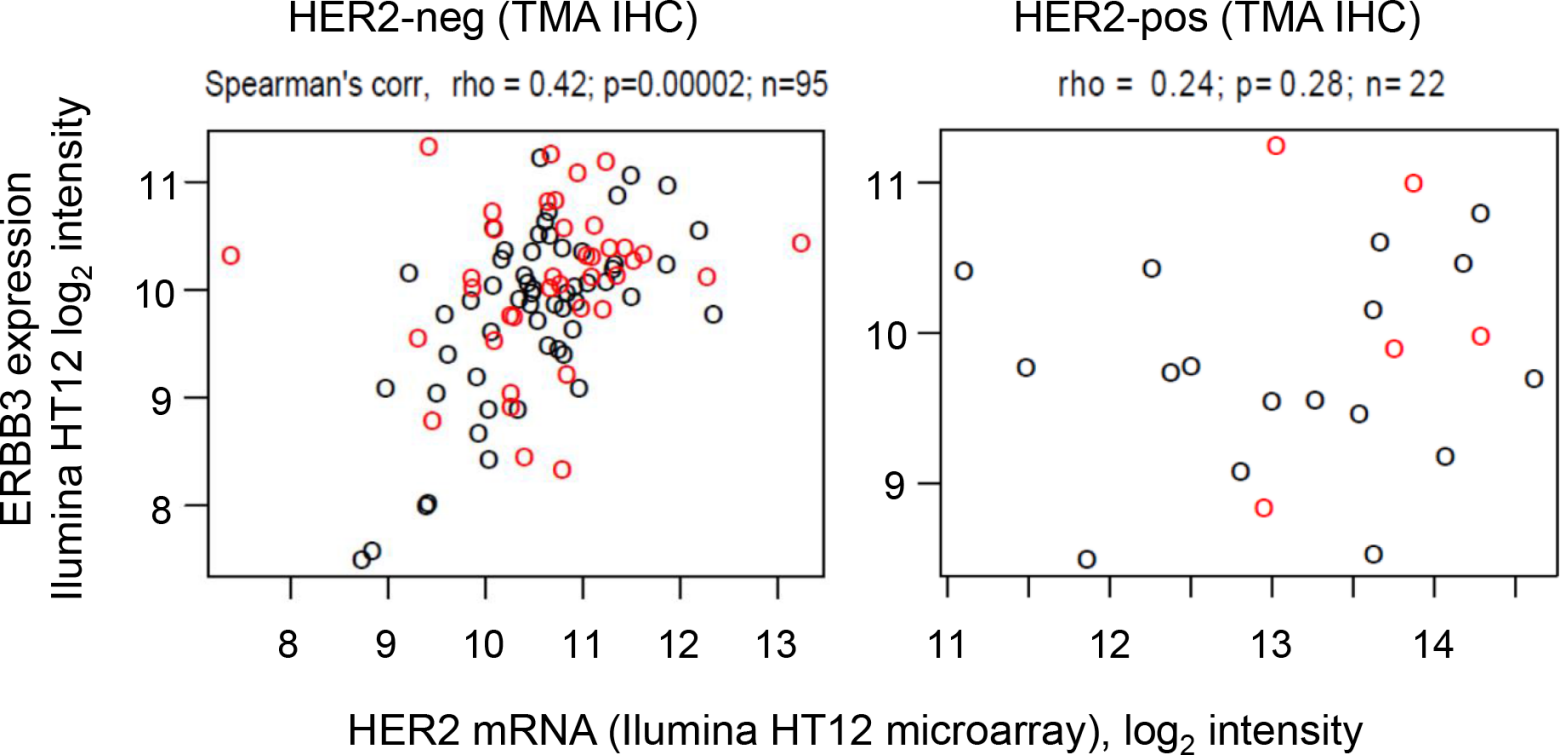
HER2 and HER3 expression levels from IIlumina HT12 microarray Data are shown for all samples with FRET imaging data for which HER2 status is available by TMA IHC (left: HER2-negative; right: HER2-positive). Points: red, FRET high; black, FRET low. The FRET efficiency threshold to define FRET high/low samples (FRET efficiency = 8.56%) was selected using an exploratory ROC curve analysis to identify an optimal dichotomization (see Methods).

In order to corroborate these findings, mRNA levels of HER2 and HER3 were assessed. Figure 3 demonstrates that mRNA levels of HER2 and HER3 were positively correlated amongst HER2 negative tumors, consistent with findings published from the MAPLE study [38]. However, samples with high FRET efficiency (red circles) were not restricted to samples with high HER2 and HER3 mRNA levels, consistent with our finding that HER2 protein levels were not correlated with HER2-HER3 dimerization as measured by FRET efficiency. These results are novel and suggest that neither HER2 and HER3 mRNA levels nor HER2 protein expression are accurate surrogates for estimating HER2-HER3 dimerization as measured by FLIM-FRET, which is based on a fundamentally stringent requirement of a < 10 nm proximity between the centres of the donor and acceptor fluorophores, that label the antibodies (Figure 1A), and provides highly specific detection of interaction.

### Mathematical models of EGFR family member interactions

The independence of HER2-HER3 interaction on their relative concentrations in the samples was a counterintuitive finding when taking into account previously published work (both experimental (HER2-HER3) and theoretical (HER2-EGFR) literature [24, 39, 40]). Although dependence of HER2-HER3 dimer on HER2 and HER3 may be expected if HER2-HER3 binding is an isolated event (e.g. *in vitro* condition), it is clear that *in vivo* HER2 and HER3 are part of a wider network of interacting EGFR family receptors consisting of at least four members (HER1-4) that are known to undergo multiple homo- and hetero-dimerization interactions in addition to HER2-HER3 binding. We therefore hypothesized that this complexity may result in a lack of correlation between HER2-HER3 dimerization and the total abundances of HER2 and HER3. In order to test this hypothesis, dynamic mathematical models of the EGFR family receptors interaction network were constructed and used to simulate and analyse the correlation of the HER2-HER3 dimer level with the total abundance of HER2 and HER3 proteins.

**Figure 4:**
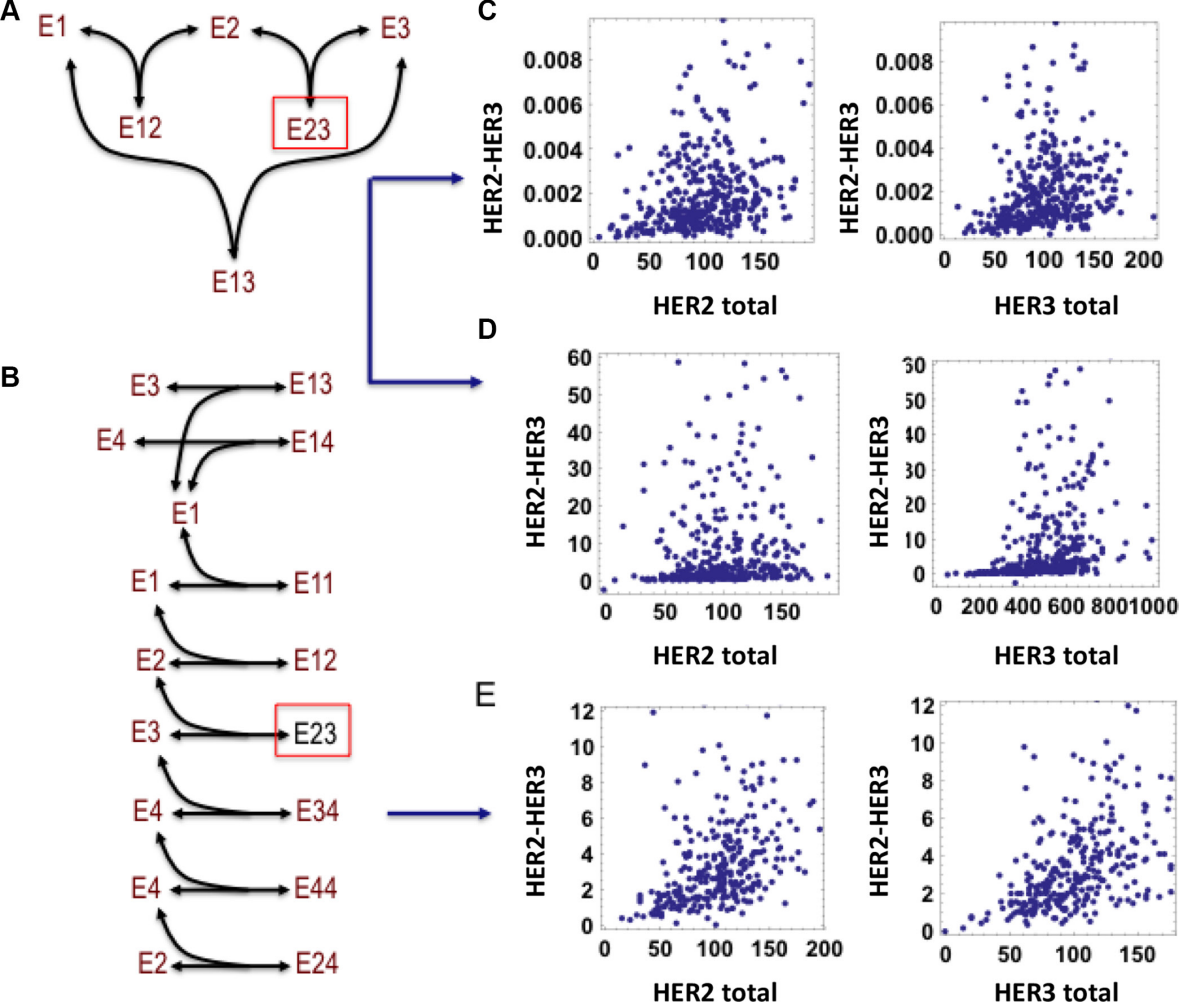
Absence of correlation between level of HER2-HER3 dimer and total HER2, HER3 abundances is revealed by mathematical modelling (**A**) Schematic diagram of a simple interaction model between HER1, HER2 and HER3. (**B**) Schematic diagram of HER1-4 interaction model with possible dimerization events including homo- and hetero-dimerization. The receptors are denoted as E1, E2, E3 and E4 for simplicity in the schemes, species Eij indicates the dimer formed between receptor Ei and Ej (the indexes i and j are between 1 and 4). (**C, D**) Lack of correlation between steady-state level of the HER2-HER3 dimer and both HER2, HER3 abundances, simulated for 400 simulated patients in the simplified model, when [HER2 total] ~ [HER3 total] << [HER1 total] (C) and [HER2 total] << [HER3 total] << [HER1 total] (D). (**E**) Lack of correlation between steady-state level of the HER2-HER3 dimer and both HER2, HER3 abundances simulated for 400 simulated patients in the detailed model, when [HER2 total] ~ [HER3 total] << [HER1 total]. Models description is given in the Supplementary Material, Tables S1–S5.

The first model, for simplicity, considered only HER2-HER3 dimerization and binding to a third receptor, in this case HER1 (Figure 4A). Simulations were run for 400 hypothetical patients where abundances of the receptors for each patient were randomly drawn from normal distributions. These commonly demonstrated a lack of correlation between HER2, HER3 and the HER2-HER3 dimer (Figure 4C, 4D). (Detailed descriptions of the model and simulation implementation are provided in the Supplementary Material).

Next the abundance of HER1 within these simulations was manipulated to examine its effect upon the correlation (Figure 4C). The higher the relative level of HER1 compared to HER2 and HER3, the poorer the correlation between HER2-HER3 dimerization and expression of HER2 or HER3. In addition, such lack of correlation is also observed if either HER2 or HER3 is significantly less expressed than the other (Figure 4D).

Conversely, if HER2 is over-expressed, becoming much more abundant than HER1 or HER3, good correlation between HER2-HER3 and HER3 was observed compared to a significant lack of correlation between HER2-HER3 and HER2 (Supplementary Figure S2B). The opposite scenario is true if HER3 becomes the over-expressed receptor (Supplementary Figure S2C). Thus, the presence of a significantly more abundant binding partner of HER2 and HER3 destroys any correlation between that receptor and its dimer(s).

The hypothesis was further tested by considering a more detailed model that consisted of all four EGFR family members (Figure 4B). The dimerization events in this model have been experimentally reported and included in previously published models [41]. Importantly, a significant lack of correlation between the levels of HER2, and HER3 and their dimer was commonly observed if similar conditions as in the reduced model in Figure 4A, were applied, i.e. if HER2 and HER3 are less abundant than HER1 (Figure 4E). The poor correlation is further amplified when HER4 is also over-expressed. Taken together, our model simulations and the analysis have confirmed our hypothesis and furthermore have teased out potential scenarios where the lack of HER2 and HER3 correlation with their dimer is common. In reality, as HER2 and HER3 also bind many other proteins outside of the EGFR family [42], it is even more likely to observe weak to no correlation between HER member overexpression and their associated dimers from experimental data.

### HER2-HER3 dimerization is associated with metastatic relapse in breast cancer patients

**Figure 5:**
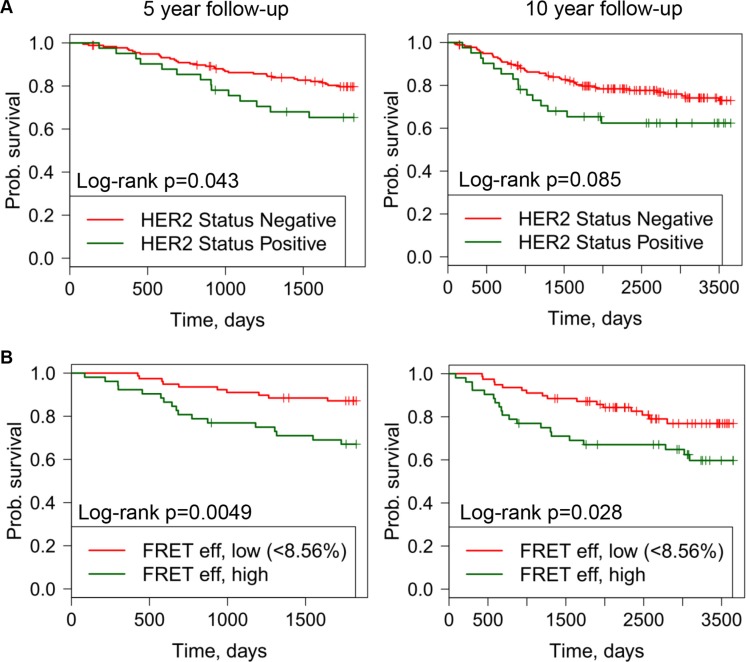
Kaplan-Meier (KM) curves off distant metastasis free survival versus FRET efficiency or HER2 status shown for follow up periods off 5 years (left) and 10 years (right) (**A**) KM plots for HER positive and negative tumor samples. (**B**) KM plots for high and low FRET efficiency.

Within the whole TMA cohort (*n* = 218), in univariate Cox survival models, HER2 status was significantly associated with metastasis-free survival at 5 years (Figure 5A). Within the cohort for which FLIM was feasible (*n* = 131) tumor samples were assigned as ‘high’ or ‘low’ FRET efficiency as a quantification of HER2-HER3 dimer formation. The FRET efficiency threshold (FRET efficiency = 8.56%) was selected using an exploratory ROC curve analysis to identify an optimal dichotomization (see Methods).

High FRET efficiency was associated with poor metastasis-free survival during a10-year follow-up period (Figure 5B). This observation was not explained by the HER2 status of the tumor samples by IHC (*p* = 0.67 by Fisher's exact test correlating HER2 IHC and FRET efficiency, Table 1). In univariate Cox survival models, additionally to high/low FRET efficiency (HR = 2.03, CI = 1.32–6.29), ER status and lymph node burden were significantly associated with metastasis-free survival during 10-year follow-up, whereas HER2 status was not found to be significantly associated in this smaller sub-cohort (*N* = 131 of the whole TMA cohort (*N* = 218), although there were only 22 (17%) HER2 IHC positive case) (Figure 5B, Supplementary Table S1). In multivariant Cox survival model high/low FRET efficiency and lymph node positivity were significantly associated with metastasis-free survival (Table 2). Furthermore, since HER2-HER3 dimer formation did not depend on the relative concentrations of HER2 protein (Figure 2C), our finding that FRET efficiency is prognostic for determining the time to distant metastasis (Figure 5B) indicates a potential new prognostic biomarker.

**Table 2:** Full multivariate Cox models of clinico-pathological and imaging data

	Multivariate; *N* = 107 with complete data			
	5 yr DMFS		10 yr DMFS	
	HR (95% C.I.)	*P*	HR (95% C.I.)	*P*
**FRET efficiency**				
High vs. Low (< 8.56%)	5.21 (1 84–14 78)	0.002	3.91 (1.61–9.5)	0.003
**HER2 status (TMAIHC)**				
Positive vs. Negative	1.12 (0.35–3.63)	0.85	0.99 (0.34–2.85)	0.99
**HER3 localisation**				
(C + M) vs. C	1.11 (0.39–3.16)	0.85	1.01 (0.4–2.57)	0.99
(M/M + C) vs. C	0.54 (0.13–2.18)	0.38	0.66 (0.22–1.98)	0.45
**ER status**				
Positive vs. Negative	0.67 (016–2 73)	0 57	0.56 (017–1 87)	0.35
**PR status**				
Positive vs. Negative	1.10 (0.33–3.75)	0.87	0.73 (0.27–2)	0.54
**Tumour size**				
> 20 mm vs. < 20 mm	1.32 (0.46–3.83)	0.61	1.32 (0.54–3.21)	0.55
**Grade**				
3 vs. (1 or 2)	1.26 (0.4–3.96)	0.69	0.62 (0.24–1.62)	0.33
**Lymph nodes positive**				
(1–3) vs. 0	3.30 (0.99–11.01)	0.05	3.20 (1.19–8.63)	0.02
(> 3) vs. 0	7.86 (1.73–35.57)	0.007	6.44 (1.73–23.97)	0.006

## DISCUSSION

The HER2-HER3 heterodimer is believed to be the most active oncogenic HER dimer, efficiently driving cellular proliferation via the PI3-kinase (PI3K)/AKT and MAPK pathways [43–45]. In this study we demonstrate the use of our recently improved FLIM analysis method [32] to derive an accurate quantification of HER2-HER3 dimer in FFPE breast samples. We show that we can readily quantify HER2-HER3 dimer formation across a range of HER2 expression scores (i.e. 0-3+, Figure 2C). As predicted from the preclinical data concerning the role of HER2-HER3 dimer in driving cellular proliferation, there was a significant association of HER2-HER3 dimer levels with recurrence of the disease (Table 2). Lymph node status was also prognostic for recurrence in this multivariate analysis, as expected. The HER2-HER3 dimer measure is a promising new biomarker to predict the likelihood of disease progression due to metastasis up to 10 years from the time of diagnosis (Figure 5B).

Interestingly, the mean proportion of HER3 that interacts with HER2 (as measured by FRET efficiency [46, 47]) does not correlate with the expression of HER2 by IHC, in contrast to that reported using PLA. At a theoretical level, we conducted simulations applying a model taking into account the complex interactions in a network of all 4 HER proteins [41] that demonstrated that a lack of correlation between HER2-HER3 dimer and HER2, HER3 levels could be commonly observed (Figure 4). Overexpression of other members of the HER family potentially interferes with the observed correlation between, e.g. HER2 overexpression and HER2-HER3 dimerization within the proposed mathematical model. This effect is likely to be more profound in biological samples due to possible interactions between HER2 and HER3 and proteins outside of the HER family. Quantification of the levels of, for instance, HER 3 and EGFR have been associated with response to targeted therapies such as lapatinib [48], or resistance to trastuzumab [49], respectively. These data suggest that dimerization between alternate members of the HER family may be more influential in predicting response to HER2 targeted treatments, than levels of HER2 alone, in support of our mathematical model.

Despite the development of efficacious targeted treatments, such as the monoclonal antibody pertuzumab, targeted against HER2-HER3 dimerization, no gold standard test has yet been validated to measure the effects of this drug on dimer formation. PLA has been successfully used on FFPE samples to quantify HER2 homodimerization and HER2-HER3 heterodimerization [23, 24]. However, it is difficult to compare precisely the distance/proximity requirement between FLIM-FRET and PLA. For PLA (primary plus secondary antibodies conjugated to oligonucleotides), the antigenic determinants recognized by two antibodies that are directly conjugated to oligonucleotides, has been estimated to be roughly 30 nm, including the dimensions of the two antibodies and the connecting oligonucleotides [25]. FRET is rarely detected beyond 1.5 × R_0_, which usually amounts to < 10 nm between the centres of donor and acceptor fluorophores) [15, 16, 18, 46, 50]. Hence, FLIM-FRET is likely to offer a more specific quantification of protein-protein interaction compared to PLA. Other techniques such as enzyme-linked immunosorbent assay (ELISA) and PCR-based techniques have been used to assess HER2 overexpression [19]. However elevated levels of HER2 extracellular domain, as measured by ELISA, were not reliably correlated with response to treatment or outcome and although PCR-based assays are sensitive and more quantitative, they do not preserve tissue morphology and samples may be contaminated by normal tissue.

Regardless of the reason for the discrepancy between the FLIM analysis and that based on the commercial PLA assay, the HER2 concentration independence could make the FLIM-based HER2-HER3 proximity parameter a useful additional marker in its utility to guide treatment decision/stratification among patients with HER2 negative tumors [51], as well as those overexpressing HER2. Some clinical studies have shown that HER2 overexpressing breast cancers are more likely to respond to lapatinib yet a small group of patients with normal or absent levels of HER2 can potentially also benefit from this treatment [52]. Other recent studies also suggest that some HER2 negative tumors can gain moderate benefit from trastuzumab treatment although robust biomarkers are needed for this indication (NSABP B-47) [53]. As we have seen, the degree of HER2-HER3 dimer formation can occur irrespective of the concentration of HER2. This heterodimer, as measured by FLIM histology, may therefore offer a new marker of HER2 dependency of the tumor (for cell proliferation, for instance) in the absence of HER2 overexpression (i.e. in HER2 negative patients). Although this study was carried out in a retrospective cohort with relatively small numbers of HER2 positive positives, further validation in a larger dataset is also planned.

## MATERIALS AND METHODS

### Antibodies

Anti-HER3 (clone B9A11, recognise intracellular epitop) was purchased from Monogram Biosciences Inc., anti-HER2 (clone e2-4001+3B5, recognise intracellular epitop) was purchased from ThermoScientific Ltd. and directly labelled according to the manufacturer's protocol with Alexa546 (X546) and Cy5, respectively.

The primary antibodies were directly labelled with fluorophore at particular dye/protein (D/P) ratio for optimal performance of the FLIM-FRET assay (donor 1:1 and acceptor 1:3), as previously described [57]. The anti-HER3 IgG (Supplementary Figure S1B) was labelled with Alexa546 (X546) (donor fluorophore) at D/P ratio ~1.0 and anti-HER2 (Supplementary Figure S1A) was labelled with Cy5 at D/P ratio ~3–4 (making it suitable as an acceptor in the assay), while retaining antigen specificity in FFPE samples.

### Plasmids

The HER3-HA construct was made by excision of HER3 from HER3-GFP (a kind gift from Selene Roberts, Rutherford Appleton Laboratory (RAL)) with NheI and KpnI, and ligation into pCDNA3.1 (Invitrogen) containing an HA tag. The construct was used for validation of anti-HER3-IgG specificity in FFPE samples (Supplementary Figure S1B).

### Cell culture and plasmid transfection

SKBR3 cells (a gift from the Breakthrough Breast Cancer Unit, Guys Hospital) and MCF-7 cells were cultured in DMEM supplemented with 10% FCS. Cells were validated by STR genotyping in ICR, UK. Cells were transfected with plasmid DNA (HER3-HA) using FuGene6 (Promega) according to the manufacturer's protocol, and cultured for 24 hours. Cells were fixed with 10% formalin for 5 hours and paraffin wax processed following a standard procedure.

### Patient samples and protein detection

Tissue microarrays (TMA) were created from 218 primary breast cancers from patients included in the METABRIC (Molecular Taxonomy of Breast Cancer International Consortium) study [58], from the King's Health Partners Cancer Biobank. Distant metastasis-free survival was defined as the time period from date of diagnosis to first distant relapse or disease-specific death. The immunohistochemical (IHC) detection of HER2 used the HercepTest™, (Dako, Glostrup, Denmark) and antigen retrieval for immunofluorescence (IF) with directly labelled antibodies was performed using the Ventana BenchMark ULTRA system, in accordance with the manufacturer's instructions.

TMA sections were cut and either stained with anti-HER3 alone (donor only) or anti-HER3 and anti-HER2 antibodies simultaneously (donor with acceptor). Expression of HER3 was assessed using the epifluorescence images from the donor only slides, prior to FLIM-FRET imaging. As there is no validated method for scoring HER3 expression, and levels vary greatly at the membrane and intra-cellularly, the presence of the receptor was scored according to the descriptive key:

M/M+C = membrane staining only or predominantly membrane, some cytoplasmic present (this combination of membrane only and membranous and cytoplasmic staining was selected due to the small numbers of cores which were scored as showing membrane staining only (*n* = 6));

C = cytoplasmic staining only

C + M = predominantly cytoplasmic staining, some occasional membrane

NA = Not Assessable

Sections were stored at −20°C until imaged.

### Imaging and image analysis

Samples were imaged on an customised “open” microscope automated FLIM system [59]. Time-domain fluorescence lifetime images were acquired via time correlated single photon counting (TCSPC) at a resolution of 256 by 256 pixels, with 256 time bins and 100 frames accumulated over 300 seconds, via excitation and emission filters suitable for the detection of Alexa546 fluorescence (Excitation filter: Semrock FF01-540/15-25; Beam Splitter: Edmund 48NT-392 30R/70T; Emission filter: Semrock FF01-593/40-25). For technical convenience, those FLIM images were acquired through the emission channel of a UV filter cube (Long pass emission filter > 420 nm). Conventional wide field fluorescence images were acquired with filter cubes for Alexa546 (Excitation 530–560 nm, emission 573–648 nm) and Cy5 (Excitation 590–650 nm, emission 663–738 nm), at a resolution of 1024 by 1024 pixels on a CCD camera (Hamamatsu 1394 ORCA-ERA), with an exposure time of typically 100–500 ms. For each sample a ‘donor’ image and a ‘donor with acceptor’ image were acquired from serial sections, of the same area of the tissue core.

FLIM analysis was performed with the TRI2 software (Version 2.7.8.9, Gray Institute, Oxford) as described previously [60, 61]. Interfering effects of autofluorescence were minimised using processing with lifetime filtering algorithm [32]. The FRET efficiency for each tissue sample region of interest was calculated according to the equation FRET eff = 1–(τ_DA_/τ_D_), where τ_D_ is the average lifetime of Alexa546 in the absence of Cy5 from the donor image and τ_DA_ is the average lifetime of Alexa546 in the presence of Cy5, from the ‘donor with acceptor’ image.

### mRNA expression

Illumina HumanHT-12 v3 Expression BeadChip data for METABRIC samples [58] were previously deposited in the European Genome-Phenome Archive (accession number EGAD00010000162). Raw probe-level data for all METABRIC samples from the King's BioBank were filtered to remove arrays with outlying low intensity (mean log2 expression < 5.6), quantile normalised using the ‘beadchip’ package for R/Bioconductor, filtered for probe detection (*p* < 0.01 on more than 1% of samples) and COMBAT corrected for beadchips.

### Statistical analysis

Kaplan-Meier curves and Cox proportional hazards models were fitted using R ‘survival’ package [62]. Univariate and full multivariate Cox models were fitted to the data. Receiver operating curves (ROC) were plotted using the ‘pROC’ package [63], using FRET as a discriminator of metastasis-free survival. From these ROC curves an optimal FRET threshold was chosen by the “Youden” method with equal weights for sensitivity and specificity, separating the patients into FRET ‘high’ and ‘low’ groups.

## CONCLUSIONS

Currently, detection of HER2 protein expression or gene amplification is central to the management of breast cancer patients and is carried out according to standardized guidelines [4]. However, the heterogeneity of HER2 expression [54], along with its ability to form heterodimers with other members of the HER family [1, 55], create a significant degree of uncertainty in reliably predicting response to treatment [54]. In support of the preclinical data pertaining to the importance of the HER2-HER3 heterodimer, clinical blockade of HER2 heterodimer formation by the addition of the novel drug, pertuzumab, to trastuzumab, has been shown to significantly improve the progression-free survival in patients with metastatic HER2+ breast cancer [56]. However, no novel biomarkers have been validated to predict response to treatment. We have demonstrated the feasibility of quantification of the HER2-HER3 heterodimer in FFPE breast tissue samples. Our assay is prognostic for metastatic recurrence up to 10 years, independent of routine clinico-pathological biomarkers, including HER2 overexpression. The assay could, if validated in a group of patients treated with, for instance pertuzumab, be used as a predictive biomarker to predict for response to such targeted therapies, in order to increase the therapeutic efficiency within the subgroup chosen and therefore also further increase the survival benefit obtained by adding pertuzumab to the trastuzumab-chemotherapy regime. The evolution of future anti-cancer treatments including the anti-HER3 agents under investigation might be enhanced by a robust, validated marker of HER2-HER3 dimerisation; in analogy to the potential role of EGFR-HER3 dimer in conferring resistance to cetuximab/panitumumab in the case of basal-like breast cancer patients [14]. By choosing the patient most likely to require and achieve a good response to anti-HER2 therapy, we can truly apply drugs where they are needed. By refining treatment via biomarker driven selection of drugs we can reduce the burden of toxicity and improve efficacy and efficiency without invariably increasing healthcare costs.

## SUPPLEMENTARY MATERIALS AND FIGURES


