# Supplementary material for: HER2-HER3 dimer quantification by FLIM-FRET predicts breast cancer metastatic relapse independently of HER2 IHC status
Source: Oncotarget. 2016 Jul 7;7(32):51012–26. doi: 10.18632/oncotarget.9963 (PMC5239455; doi:10.18632/oncotarget.9963)
Supplement: Supplementary file 1 [file oncotarget-07-51012-s001.pdf]

# HER2-HER3 dimer quantification by FLIM-FRET predicts breast cancer metastatic relapse independently of HER2 IHC status

## Supplementary Materials

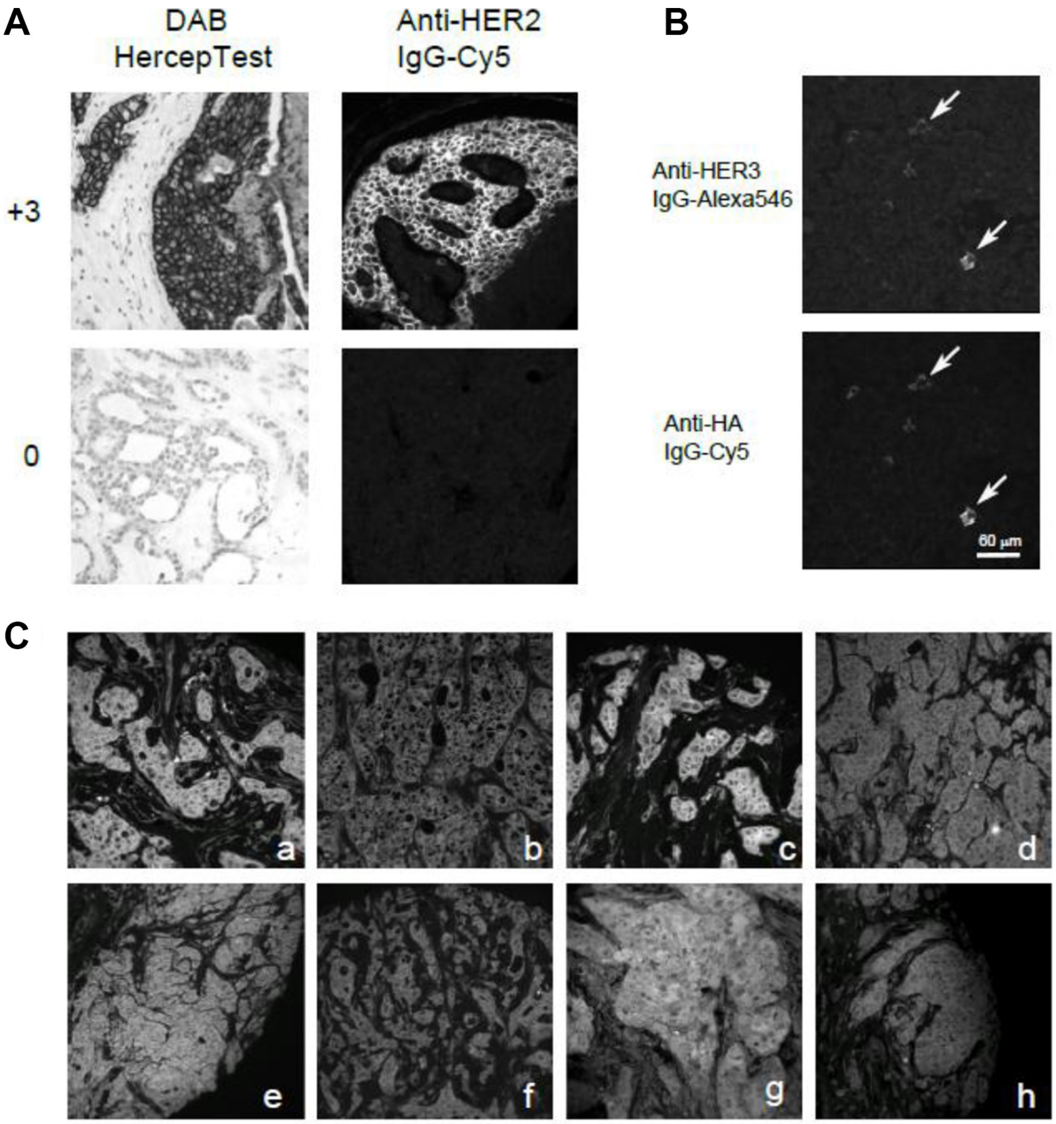


**Supplementary Figure S1:** (**A**) Comparison of pattern of staining between anti-HER2-IgG-Cy5 and HercepTest DAB staining in 3+ and 0+ scored tissues. (**B**) Validation of anti-HER3-IgG-Alexa546 specificity in FFPE MCF-7 cells overexpressing HER3-HA. Co-localisation of anti-HER3-IgG-Alexa546 and anti-HA-Cy5 in cells overexpressing HER3-HA (white arrows) indicative of high specificity of antibody. (**C**) Representative images of tissue stained with anti-HER3-IgG-Alexa546 showing different pattern of staining (predominantly membrane, a-d, vs. cytoplasm, e-h) and different intensity (a, c, e, g vs. b, d, f, h).

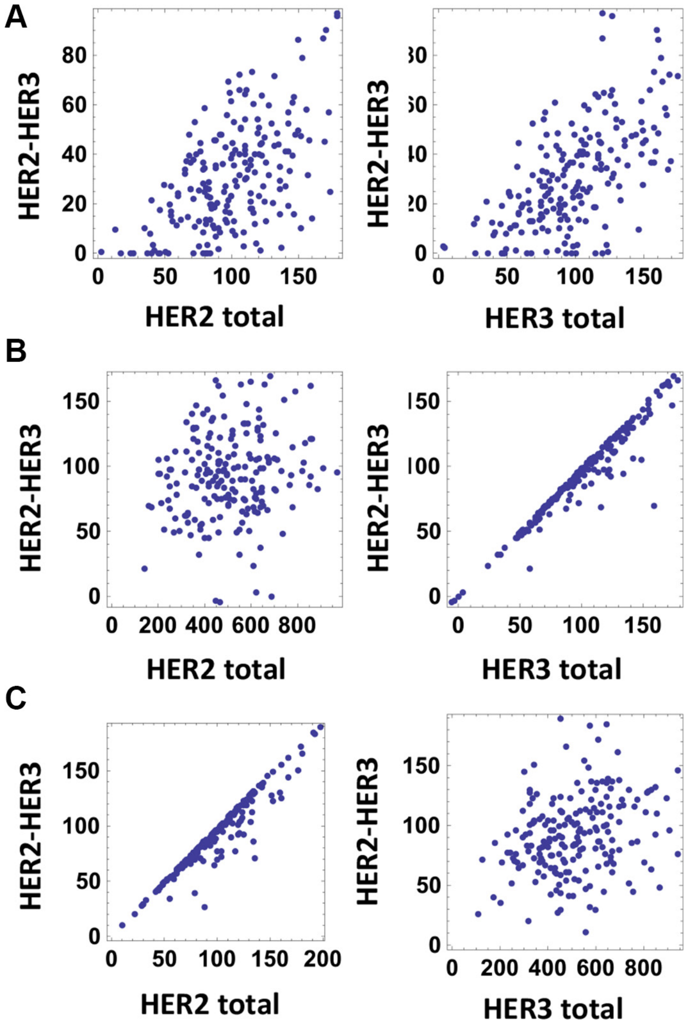


**Supplementary Figure S2: Scatter plots of simulated HER2-HER3 dimer and total HER2, HER3 abundances in different conditions in the simple model.** (**A**) [HER2 total] = [HER3 total] = [HER1 total], and the HER2-HER3 binding affinity is relatively weaker than HER1-HER2 and HER1-HER3 bindings. Some although weak correlations are observed. (**B**) [HER1 total] = [HER3 total] << [HER2 total]. When HER2 is more abundant (over-expressed) than the remaining receptors, no correlation is observed for HER2-HER3 and HER2 but strong correlation seen for HER2-HER3 and HER3. (**C**) As in (B) but in this case HER3 is the more abundant receptor.

## Supplementary Table S1: Univariate Cox survival model analysis for 131 patient's samples available for FRET-FLIM analysis

| | Univariate | | | |
|---|---|---|---|---|
| | 5yr DMFS | | 10yr DMFS | |
| | HR (95% C.I.) | *P* | HR (95% C.l.) | *P* |
| **FRET efficiency** | | | | |
| High vs. Low (< 8.56%) | 2.88 [1.32–6.29) | 0.01 | 2.03 (1.05–3.92) | 0.035 |
| **HER2 status (TMA IHC)** | | | | |
| Positive vs. Negative | 1.24 [0.46–3.37) | 0.67 | 1.03 (0.42–2.51) | 0.95 |
| **HER3 Iocalisation** | | | | |
| (C + M) vs. C | 1.29 [0.56–2.98) | 0.55 | 0.95 (0.49–2.27) | 0.89 |
| (M/M + C) vs. C | 0.49 [0.14–1.72) | 0.27 | 1.73 (0.22–1.55) | 0.28 |
| **ER status** | | | | |
| Positive vs. Negative | 0.39 [0.17–0.89) | 0.02 | 0.38 (0.18–0.8) | 0.01 |
| **PR status** | | | | |
| Positive vs. Negative | 0.57 [0.27–1.21) | 0.14 | 0.55 (0.28–1.05) | 0.07 |
| **Tumour size** | | | | |
| > 20 mm vs. < 20 mm | 2.06 [0.87–4.86) | 0.10 | 1.86 [0.9–3.86) | 0.09 |
| **Grade** | | | | |
| 3 vs. [1 or 2) | 1.70 (0.77–3.75) | 0.19 | 1.07 (0.55–2.08) | 0.34 |
| **Lymph nodes positive** | | | | |
| (1–3) vs. 0 | 3.23 (1.06–9.82) | 0.04 | 3.20 (1.29–7.98) | 0.01 |
| (> 3) vs. 0 | 6.99 (2.15–22.72) | 0.001 | 5.36 (1.95–14.77) | 0.001 |

### 1. Development of a simple interaction model consisting of HER1, HER2 and HER3

We employed a modelling approach based on ordinary differential equations (ODE). The reaction rates were formulated generally at the elementary step level, using mass-action kinetic law. The kinetic scheme of the model is given in Figure 3A (main text) while description of the model reactions, ODE equations and parameters is given in Table S2 and S3 below, based on which any reader can reconstruct the model in a straightforward manner. In addition, the *Mathematica* code of the model can be provided upon request.

## Supplementary Table S2: Reactions and reaction rates of the HER1-3 interaction model presented in Figure 4A

| No | Reactions | Reaction rates | Reference parameter values | References |
|---|---|---|---|---|
| **1** | E1 + E2 ↔ E12 | **v1** = kf1*[E1]*[E2] – kr1*[E12] | kf1 = 0.0001<br>kr1 = 0.001 | (Birtwistle et al. MSB 2007) |
| **2** | E2 + E3 ↔ E23 | **v2** = kf2*[E3]*[E2] – kr2*[E23] | kf2 = 0.0001<br>kr2 = 0.001 | (Birtwistle et al. MSB 2007) |
| **3** | E1 + E3 ↔ E13 | **v3** = kf3*[E1]*[E3] – kr3*[E13] | kf3 = 0.0001<br>kr3 = 0.001 | (Birtwistle et al. MSB 2007) |

The first- (dissociation $k_r$, catalytic $k_c$, degradation $k_d$) and second-order (association $k_f$) rate constants are expressed in $s^{-1}$ and $nM^{-1} s^{-1}$. Synthesis rate is expressed in $nM\ s^{-1}$. Model parameters are either measured or primarily based on our previous publication (43). Note that HER1, 2, 3 are denoted as E1, E2 and E3 respectively for ease of reference.

## Supplementary Table S3: Ordinary differential equations of the HER1-3 interaction model

| Left-hand Sides | Right-hand Sides | Reference initial Concentrations (nM) | Based on references |
|---|---|---|---|
| d[E1]/dt | – v1 – v3 | 100 | (Birtwistle et al. MSB 2007) |
| d[E2]/dt | – v1 – v2 | 100 | (Birtwistle et al. MSB 2007) |
| d[E3]/dt | – v2 – v3 | 100 | (Birtwistle et al. MSB 2007) |
| d[E12]/dt | v1 | 0 | |
| d[E23]/dt | v2 | 0 | |
| d[E13]/dt | v3 | 0 | |

The reaction rates are given in Table S1

### 2. Development of a detailed interaction model consisting of all four ErbB family receptors HER1 – HER4.

Similarly, the model is constructed using ODEs and formulated by mass-action kinetic law. The kinetic scheme of the model is given in Figure 3B (main text) while description of the model reactions, ODE equations and parameters is given in Table S4 and S5 below, based on which any reader can reconstruct the model in a straightforward manner. In addition, the *Mathematica* code of the model can be provided upon request.

## Supplementary Table S4: Reactions and reaction rates of the HER1-4 interaction model presented in Figure 4B

| No | Reactions | Reaction rates | Reference parameter values | References |
|---|---|---|---|---|
| **1** | E1 + E2 ↔ E12 | **v1** = kf1*[E1]*[E2] – kr1*[E12] | kf1 = 0.0001<br>kr1 = 0.001 | (Birtwistle et al. MSB 2007) |
| **2** | E2 + E3 ↔ E23 | **v2** = kf2*[E3]*[E2] – kr2*[E23] | kf2 = 0.0001<br>kr2 = 0.001 | (Birtwistle et al. MSB 2007) |
| **3** | E1 + E3 ↔ E13 | **v3** = kf3*[E1]*[E3] – kr3*[E13] | kf3 = 0.0001<br>kr3 = 0.001 | (Birtwistle et al. MSB 2007) |
| **4** | E1 + E4 ↔ E14 | **v4** = kf4*[E1]*[E4] – kr4*[E14] | kf4 = 0.0001<br>kr4 = 0.001 | (Birtwistle et al. MSB 2007) |
| **5** | E1 + E1 ↔ E11 | **v5** = kf5*[E1]*[E1] – kr5*[E11] | kf5 = 0.0001<br>kr5 = 0.001 | (Birtwistle et al. MSB 2007) |
| **6** | E3 + E4 ↔ E34 | **v6** = kf6*[E4]*[E3] – kr6*[E34] | kf6 = 0.0001<br>kr6 = 0.001 | (Birtwistle et al. MSB 2007) |
| **7** | E4 + E4 ↔ E44 | **v7** = kf7*[E4]*[E4] – kr6*[E44] | kf7 = 0.0001<br>kr7 = 0.001 | (Birtwistle et al. MSB 2007) |
| **8** | E2 + E4 ↔ E24 | **v8** = kf8*[E4]*[E2] – kr7*[E24] | kf8 = 0.0001<br>kr8 = 0.001 | (Birtwistle et al. MSB 2007) |

The first- (dissociation $k_r$, catalytic $k_c$, degradation $k_d$) and second-order (association $k_f$) rate constants are expressed in $s^{-1}$ and $nM^{-1}$ $s^{-1}$. Synthesis rate is expressed in nM $s^{-1}$. Model parameters are either measured or primarily based on our previous publication (55). Note that HER1, 2, 3 are denoted as E1, E2 and E3 respectively for ease of reference.

## Supplementary Table S5: Ordinary differential equations of the HER1-4 interaction model

| Left-hand Sides | Right-hand Sides | Reference initial Concentrations (nM) | Based on references |
|---|---|---|---|
| d[E1]/dt | – v1 – v5 – v3 – v4 | 100 | (Birtwistle et al. MSB 2007) |
| d[E2]/dt | – v1 – v2– v8 | 100 | (Birtwistle et al. MSB 2007) |
| d[E3]/dt | – v2 – v6 – v3 | 100 | (Birtwistle et al. MSB 2007) |
| d[E4]/dt | – v6 – v7 – v4 | 100 | (Birtwistle et al. MSB 2007) |
| d[E13]/dt | v3 | 0 | |
| d[E14]/dt | v4 | 0 | |
| d[E11]/dt | v5 | 0 | |
| d[E12]/dt | v1 | 0 | |
| d[E23]/dt | v2 | 0 | |
| d[E34]/dt | v6 | 0 | |
| d[E44]/dt | v7 | 0 | |
| d[E24]/dt | v8 | 0 | |

The reaction rates are given in Table S3.

### 3. Model Simulations

To investigate possible correlation between the steady state level of HER2-HER3 (E23) dimer and the steady state abundances of HER2 and HER3, we first assume typical values of the kinetic constant rates for the reactions of both models in Figure 4 and B following (55) as detailed in Tables S2–S5. Since the abundances of HER1-4 can significantly vary from patient to patient, in order to simulate what may happen in an ensemble of patients, we randomly draw these abundances from normal distributions with means being the reference values given in Tables S1–S4, and standard deviation = mean/fold, where fold =3. We then simulate the model to equilibrium and compute the steady-state level of the HER2-HER3 dimer for each *in silico* patient. All plots were produced with a cohort size of 400 patient samples, comparable with the number of samples in our experimental study. Note that varying the fold value did not affect our conclusions.

Concentration for over-expressed receptors was assumed to be 500–1000 nM, which is 5–10 times larger than the reference values. For example, over-expressed HER1 in the simple and detailed models were assumed to be 1000 nM. When investigating effect of varying the affinity of a binding reaction, we typically reduced its dissociation constant (Kd) by 10–100 times by increasing the association rate kf by 10–100 times the reference values given in Tables S2–S5.
